# Supplementary material for: General variation in the Fusarium wilt rhizosphere microbiome
Source: Nat Commun. 2025 Dec 27;17:1017. doi: 10.1038/s41467-025-67760-y (PMC12847888; doi:10.1038/s41467-025-67760-y)
Supplement: Supplementary file 1 — Supplementary Information [file 41467_2025_67760_MOESM1_ESM.pdf]

General variation in the *Fusarium* wilt rhizosphere microbiome

Table S1 Plant types and sample numbers

Plant	Sample Number	Sequencing Data
Watermelon	36	Amplicon sequencing
Cucumber	8	Amplicon sequencing
Panax	48	Amplicon sequencing
Pea	72	Amplicon sequencing
Pepper	12	Amplicon sequencing
Avocado	10	Amplicon sequencing
Tomato	12	Amplicon sequencing
Panax	12	Metagenomic sequencing
Zanthoxylum	10	Metagenomic sequencing

Table S2 Meta-information of OAUs (operational analysis units)

OAU_ID	Latitude	Longitude	Treatment	Project	Sample Numbers	Study title
OAU1	33.07	117.04	NULL	PRJNA689051	10	Shifts in the structure of rhizosphere bacterial communities of avocado after *Fusarium* dieback
OAU2	49.46	-107.14	NULL	PRJNA562911	8	Root rot alters the root-associated microbiome of field pea in commercial crop production systems
OAU3	50.12	-107.44	NULL	PRJNA562911	8	
OAU4	50.15	-106.58	NULL	PRJNA562911	8	
OAU5	49.49	-107.5	NULL	PRJNA562911	8	
OAU6	49.23	-104.32	NULL	PRJNA562911	8	
OAU7	49.26	-104.35	NULL	PRJNA562911	8	
OAU8	50.23	-107.5	NULL	PRJNA562911	8	
OAU9	51.1	-107.43	NULL	PRJNA562911	8	
OAU10	50.1	-108.16	NULL	PRJNA562911	8	
OAU11	24.12	102.75	NULL	PRJNA588201	18	Probiotic consortia: Reshaping the rhizospheric microbiome and its role in suppressing root-rot disease of *Panax notoginseng*
OAU12	25.46	112.43	NULL	PRJNA667302	6	Disease-induced changes in plant microbiome assembly and functional adaptation
OAU13	26.07	106.24	NULL	PRJNA667302	6	
OAU14	126.19	41.16	two years	PRJNA634905	8	Outbreaks of root rot disease in different aged American ginseng plants are associated with field microbial dynamics
OAU15	126.19	41.16	three years	PRJNA634905	8	
OAU16	126.19	41.16	four years	PRJNA634905	8	
OAU17	28.48	113.35	BOF_Dazomet_5	PRJNA664653	6	Microbial assembly and association network in watermelon rhizosphere after soil fumigation for *Fusarium* wilt control
OAU18	28.48	113.35	Dazomet_5	PRJNA664653	6	
OAU19	28.48	113.35	Dazomet_6	PRJNA664653	6	
OAU20	28.48	113.35	BOF_Dazomet_6	PRJNA664653	6	
OAU21	28.48	113.35	BOF_Dazomet_8	PRJNA664653	6	
OAU22	28.48	113.35	Dazomet_8	PRJNA664653	6	
OAU23	33.66	106.72	NULL	PRJNA494298	6	Changes in the soil microbial community are associated with the occurrence of *Panax quinquefolius* L. root rot diseases
OAU24	35.23	114.37	NULL	PRJNA625866	8	Exploring the communities of bacteria, fungi and ammonia oxidizers in rhizosphere of *Fusarium*-diseased greenhouse cucumber
OAU25	36.92	118.77	NULL	PRJNA485233	12	Changes in bacterial and fungal microbiomes associated with tomatoes of healthy and infected by *Fusarium oxysporum* f. sp. *lycopersici*

Table S3 Identification of microbial biomarkers in diseased and healthy root microbiomes using random forest in mlr framework

Genus	Importance	*P*	*P*_adj
Solirubrobacter	4.72	0.8520	0.8520
Stenotrophomonas	3.472	0.0050	0.0110
Sporocytophaga	3.421	0.4420	0.5402
Stella	3.337	0.5680	0.6248
Microvirga	3.317	0.0240	0.0440
Variovorax	3.075	0.0001	0.0011
Luteimonas	2.516	0.1980	0.2723
Flavobacterium	2.364	0.0020	0.0073
Sphingobacterium	2.323	0.0470	0.0739
Aeromicrobium	2.276	0.0010	0.0055
Taibaiella	2.229	0.0040	0.0110

Note: Statistical significance was determined based on a linear mixed effects model. *P* values were adjusted by Benjamini-Hochberg FDR correction. The analyses were performed on n = 93 and 105 for diseased and healthy independent rhizosphere samples.

Table S4 The functions of predictors

Predictor	Functions	Study title	References
Variovorax	Stimulate plant growth	(1) Transcriptome profiling of *Variovorax* paradoxus EPS under different growth conditions reveals regulatory and structural novelty in biofilm formation<br>(2) A single bacterial genus maintains root growth in a complex microbiome	Fredendall et al. (2020) Access Microbiol<br>Finkel et al. (2020) Nature
Flavobacterium	Stimulate plant growth and inhibit *Fusarium* growth	(1) Biochemical characterization and antifungal activity of a recombinant β-1,3-glucanase FlGluA from *Flavobacterium* sp. NAU1659<br>(2) *Flavobacterium plantiphilum sp. nov., Flavobacterium rhizophilum sp. nov., Flavobacterium rhizosphaerae sp. nov., Chryseobacterium terrae sp. nov., and Sphingomonas plantiphila sp. nov.* isolated from salty soil showing plant growth promoting potential	Wang et al. (2024) Protein Expr Purif<br>Kämpfer et al. (2025) Syst Appl Microbiol
Stenotrophomonas	Stimulate plant growth and inhibit *Fusarium* growth	(1) Volatile organic compounds from *Stenotrophomonas geniculata* J-0 as potential biofumigants manage bulb rot caused by *Fusarium oxysporum* in postharvest Lanzhou lily<br>(2) Evidence for the plant recruitment of beneficial microbes to suppress soil-borne pathogens	Ling et al. (2024) World J Microbiol Biotechnol<br>Liu et al. (2021) New Phytol
Rhizobium	Stimulate plant growth	Rhizobia: A promising source of plant growth-promoting molecules and their non-legume interactions: examining applications and mechanisms	Fahde et al. (2023) Agriculture
Williamsia	Opportunistic pathogen	*Williamsia muralis gen.* nov., sp. nov., isolated from the indoor environment of a children's day care centre	Kämpfer et al. (1999) Int J Syst Bacteriol
Novosphingobium	Degrade allelochemicals/lignin	(1) Metagenomics insights into responses of rhizobacteria and their alleviation role in licorice allelopathy<br>(2) Laboratory evolution in *Novosphingobium aromaticivorans* enables rapid catabolism of a model lignin-derived aromatic dimer	Liu et al. (2023) Microbiome<br>Allemann et al. (2025) Appl Environ Microb
Micromonospora	Stimulate plant growth and inhibit *Fusarium* growth	(1) *Micromonospora*: An important microbe for biomedicine and potentially for biocontrol and biofuels<br>(2) Antagonistic bacteria against *Fusarium* spp. isolated from sclerotia of Claviceps gigantea in maize (Zea mays)	Hirsch and Valdés (2010) Soil Boil Biochem<br>Ayala-Torres et al. (2023) Rev. mex. fitopatol

Table S5 Functions of the KEGG ortholog in published papers

KO	Functions	Function group	Study title
K03564	thioredoxin-dependent peroxiredoxin	Antioxidant functions	A novel thioredoxin-dependent peroxiredoxin (TPx-Q) plays an important role in defense against oxidative stress and is a possible drug target in *Babesia microti*
K00566	tRNA-uridine 2-sulfurtransferase	Sulfur transport	Trafficking in persulfides: delivering sulfur in biosynthetic pathways
K21147	sulfur-carrier protein adenylyltransferase	Sulfur transport	The functional diversity of the prokaryotic sulfur carrier protein TusA
K03636	sulfur-carrier protein	Sulfur transport	The functional diversity of the prokaryotic sulfur carrier protein TusA
K00029	malate dehydrogenase	Carbon metabolism	Association of the malate dehydrogenase-citrate synthase metabolon is modulated by intermediates of the Krebs tricarboxylic acid cycle
K01687	dihydroxy-acid dehydratas	Carbon metabolism	Characterization of recombinantly expressed dihydroxy-acid dehydratase from *Sulfobus solfataricus*—A key enzyme for the conversion of carbohydrates into chemicals
K01207	degradation of chitin acetylhexosaminidase	Carbon metabolism	Characterization of β-N-acetylhexosaminidase (LeHex20A), a member of glycoside hydrolase family 20, from *Lentinula edodes* (shiitake mushroom)
K04763	integrase	Adverse stress	Prevalence of SOS-mediated control of integron integrase expression as an adaptive trait of chromosomal and mobile integrons
K07636	phosphate regulon sensor histidine kinase	Adverse stress	The phosphate regulon and bacterial virulence: a regulatory network connecting phosphate homeostasis and pathogenesis
K17759	NAD(P)H-hydrate dehydratase	Adverse stress	NAD(P)H-hydrate dehydratase- a metabolic repair enzyme and its role in *Bacillus subtilis* stress adaptation

Table S6. Significantly coenriched genes in the diseased rhizosphere from the metagenome analysis of different OAUs

KO	Function	Dis	Hea	*P*	*P*_adj	Fold (D/H)	OAU
K00012	UDPglucose 6-dehydrogenase	367.8±34.41	359.12±8.21	0.0243	0.1417	1.02	OAU1
		377.33±8.35	312.58±54.16	0.0324	0.6407	1.21	OAU2
K01207	beta-N-acetylhexosaminidase	296.23±2.83	263.73±22.09	0.0005	0.0222	1.12	OAU1
		265.89±35.9	232.13±28.64	0.0301	0.6407	1.15	OAU2
K01754	threonine dehydratase	596.13±36.23	584.2±13.07	0.0225	0.1379	1.02	OAU1
		558.57±36.55	488.66±56.07	0.027	0.6407	1.14	OAU2
K00566	tRNA-uridine 2-sulfurtransferase	241.74±22.59	236.16±9.36	0.0441	0.1976	1.02	OAU1
		189.26±32.32	164.52±30.58	0.0014	0.3421	1.15	OAU3
K03564	thioredoxin-dependent peroxiredoxin	360.69±23.12	329.19±8.87	0.0011	0.0367	1.1	OAU1
		401.72±97.34	267.18±69.57	0.0245	0.6407	1.50	OAU2
K03636	sulfur-carrier protein	252.93±43.47	198.77±17.05	0.0001	0.0094	1.27	OAU1
		295.88±77.48	207.71±32.01	0.0371	0.6407	1.42	OAU2
K05710	trans-cinnamate dioxygenase ferredoxin component	192.98±20.17	177.83±17.46	0.0228	0.1379	1.09	OAU1
		265.44±104.17	156.54±69.77	0.0416	0.6407	1.70	OAU2
K21147	sulfur-carrier protein adenylyl transferase	246.83±24.48	228.97±25.31	0.0283	0.1530	1.08	OAU1
		214.37±47.29	182.69±34.23	0.0459	0.9993	1.17	OAU3
K04763	integrase/recombinase	374.98±18.76	366.53±19.32	0.0429	0.1945	1.02	OAU1
		463.79±117.71	326.01±84.98	0.048	0.6407	1.42	OAU2
K07636	phosphate regulon sensor histidine kinase	348.11±20.71	308.85±25.66	0.0007	0.0261	1.13	OAU1
		287.11±46.43	261.6±39.91	0.0174	0.9993	1.10	OAU3
K17758	ADP-dependent NAD(P)H-hydrate dehydratase	222.13±17.84	198.12±15.45	0.002	0.0411	1.12	OAU1
		204.51±31.09	129.12±56.26	0.0377	0.6407	1.58	OAU2
K17759	NAD(P)H-hydrate epimerase	215.21±19.58	189.03±13.67	0.0011	0.0367	1.14	OAU1
		199.51±30.75	120.15±60.93	0.0442	0.6407	1.66	OAU3

Note: Operational analysis unit (OAU) represents a subgroup from a study with multiple treatments, with the aim of comparing healthy and diseased samples under the same conditions. OAU1 represents *Zanthoxylum* plants from 103°75′E_31°77′N, OAU2 represents *Panax* plants from 100°3′E_ 26°49′N, and OAU3 represents *Panax* plants from 103°68′E_ 24°2′N. Statistical significance was determined using the DESeq2 package. *P* values were adjusted by Benjamini-Hochberg FDR correction. The analyses were performed on n = 11 for independent rhizosphere samples.

Table S7. Significantly coenriched genes in the healthy rhizosphere from the metagenome analysis of different OAUs

KO	Function	Dis	Hea	*P*	*P*adj	Fold (D/H)	OAU
K00029	malate dehydrogenase	190.4±23.19	259.09±38.5	0.0020	0.0411	0.73	OAU1
		221.48±73.87	342.42±84.54	0.0406	0.6407	0.65	OAU2
K00249	acyl-CoA dehydrogenase	931.63±53.51	1156.43±136.45	0.0044	0.0588	0.81	OAU1
		906.58±221.48	1315.06±314.93	0.0442	0.6407	0.69	OAU2
K00574	cyclopropane-fatty-acyl-phospholipid synthase	226.2±24.32	281.58±43.13	0.048	0.2062	0.80	OAU1
		212.12±53.97	361.39±121.88	0.016	0.6407	0.59	OAU2
K00831	phosphoserine aminotransferase	144.14±15.74	200.41±21.98	0	0.0056	0.72	OAU1
		113.48±22.23	214.71±96.72	0.0141	0.6407	0.53	OAU2
K01251	adenosyl homocysteinase	365.79±46.24	458.21±22.91	0.0019	0.0411	0.80	OAU1
		326.31±64.83	418.33±19.27	0.0347	0.6407	0.78	OAU2
K01626	3-deoxy-7-phosphoheptulonate synthase	172.12±18.58	229.06±31.54	0.0458	0.0458	0.75	OAU1
		140.16±29.3	254.93±126.22	0.03	0.6407	0.55	OAU2
K01679	fumarate hydratase	255.62±35.69	311.71±26.48	0.0263	0.1490	0.82	OAU1
		193.27±44.71	221.32±38.4	0.001	0.3348	0.87	OAU3
K01687	dihydroxy-acid dehydratase	456.26±45.12	567.64±50.14	0.0078	0.0837	0.80	OAU1
		481.64±113.09	665.61±97.06	0.0353	0.6407	0.72	OAU2
K03781	catalase	175.49±30.82	264.62±36.1	0	0.0056	0.66	OAU1
		131.53±37.74	252.4±149.82	0.0367	0.6407	0.52	OAU2
K03782	catalase-peroxidase	168.02±18.03	238.03±40.82	0.0002	0.0113	0.71	OAU1
		145.62±25.76	168.08±15.5	0.0037	0.7268	0.87	OAU3
K07716	two-component system, cell cycle sensor histidine kinase	147.58±30.2	191.43±9.25	0.0313	0.1572	0.77	OAU1
		141.84±57.22	232.44±36.45	0.0231	0.6407	0.61	OAU2
K21470	L,D-transpeptidase	169.25±8.05	209.44±17.3	0.0193	0.1248	0.81	OAU1
		109.52±50.95	198.49±40.45	0.0206	0.6407	0.55	OAU2
K03694	ATP-dependent Clp protease ATP-binding subunit	243.38±34.79	335.67±32.12	0.0002	0.0118	0.73	OAU1
		219.41±66.41	313.64±51.29	0.0355	0.6407	0.70	OAU2

Note: OAU1 represents Zanthoxylum plants from 103°75′E_31°77′N, OAU2 represents Panax plants from 100°3′E_ 26°49′N, and OAU3 represents *Panax* plants from 103°68′E_ 24°2′N. Statistical significance was determined using a linear mixed effects model with the formula: relative abundance ~ health_status + (1 | OAU), followed by ANOVA. Statistical significance was determined using the DESeq2 package. *P* values were adjusted by Benjamini-Hochberg FDR correction. The analyses were performed on n =11 for diseased and healthy independent rhizosphere samples.

Table S8 Genes shared between the *F. anhuiense* K5 genome and the metagenome in the rhizosphere

KO	Functions	Copy numbers
K01207	beta-N-acetylhexosaminidase	2
K00566	tRNA-uridine 2-sulfurtransferase	1
K03564	thioredoxin-dependent peroxiredoxin	2
K07636	phosphate regulon sensor histidine kinase	2
K04763	integrase/recombinase XerD	1
K00012	UDP glucose 6-dehydrogenase	1

Table S9 The significantly enriched root exudates components in healthy tomato plants

Root exudates	CK	FS	*P*	$P_{\_adj}$
myo-inositol	25.8±2.36	22.25±0.83	0.0295	0.1053
2-(1-methyl-2-pyrrolidinyl)-pyridine	16.75±3.21	10.68±0.63	0.0099	0.0381
monomyristin	14.3±0.99	9.03±0.15	0.0000	0.0004
N-acetylglycine	11.64±0.59	10.79±0.2	0.0327	0.1089
trehalose-6-phosphate	12.58±0.8	9.82±0.17	0.0005	0.0026
hexitol	12.42±0.81	9.74±0.22	0.0007	0.0028
n-acetyl-d-hexosamine 1	17.53±11.53	2.19±3.99	0.0457	0.1269
2,4-dichloro-1-(2-chloroethenyl)-benzene	8.51±0.57	7.74±0.11	0.0378	0.1136
d7-glucose	8.21±0.46	5.64±0.35	0.0001	0.0007
N-acetylputrescine	8.21±0.46	5.64±0.35	0.0001	0.0007
xylonolactone	8.21±0.46	5.64±0.35	0.0001	0.0007
homoserine	7.74±0.52	3.6±0.1	0.0000	0.0001

Statistical significance was determined using a two-sided unpaired t-test. *P* values were adjusted by Benjamini-Hochberg FDR correction ($n = 4$ biologically independent samples per group).

Table S10 Comparison of the relative abundances of the dominant genera (top 15) in the soil between the control and tocopherol acetate treatments. C and T represent the control and tocopherol acetate treatments, respectively.

Genus	Relative abundances (%)		*P*	*P*_adj
	C	T		
Flavobacterium	5.19±1.92	1.18±0.32	0.0000	0.0000
Pedomicrobium	0.04±0.01	0.06±0.02	0.0226	0.3916
Symbiobacterium	0.05±0.02	0.07±0.01	0.0235	0.3916
Cystobacter	0.18±0.04	0.23±0.05	0.0649	0.5514
Micromonospora	0.28±0.03	0.25±0.03	0.0603	0.5514
Nonomuraea	0.14±0.03	0.11±0.03	0.0708	0.5514
Phenylobacterium	0.08±0.02	0.11±0.01	0.0772	0.5514
Cohnella	0.06±0.02	0.08±0.02	0.0972	0.6073
Conexibacter	0.23±0.04	0.27±0.03	0.1652	0.6471
Mycobacterium	0.21±0.04	0.18±0.03	0.1175	0.6471
Nocardioides	2.16±0.2	2.03±0.08	0.1493	0.6471
Phycicoccus	0.07±0.02	0.06±0.01	0.1594	0.6471
Streptomyces	2.01±0.09	1.92±0.13	0.1682	0.6471
Zavarzinella	0.09±0.01	0.11±0.03	0.2347	0.8381
Aeromicrobium	0.06±0.03	0.08±0.02	0.4372	0.8888

Statistical significance was determined using the DESeq2 package. *P* values were adjusted by Benjamini-Hochberg FDR correction ($n = 5$ biologically independent samples per group).

Table S11 Relative expression levels of significantly upregulated genes (top 15 genes with the greatest abundance) in *F. anhuiense* K5 between the control and tocopherol acetate treatments. C and T represent the control and tocopherol acetate treatments, respectively.

Gene Id	Relative expression (FPKM)		Fold	Functinos	*P*	*P*_adj
	C	T				
gene3467	21957.76	78103.09	3.56	MULTISPECIES: YceI family protein	6.27E-21	8.98E-20
gene2192	8445.78	28085.06	3.33	MULTISPECIES: NADP-dependent glyceraldehyde-3-phosphate dehydrogenase	7.8E-154	3.7E-151
gene272	3563.06	27451.68	7.70	secretion protein	0	0
gene3468	5439.44	22759.69	4.18	MULTISPECIES: hypothetical protein	2.1E-99	4.68E-97
gene3848	6763.40	19854.43	2.94	biosynthetic-type acetolactate synthase large subunit	4.67E-71	4.5E-69
gene3852	7269.82	16268.49	2.24	MULTISPECIES: 2-isopropylmalate synthase	3.27E-74	3.65E-72
gene4131	7155.47	15965.54	2.23	L-aspartate oxidase	2.39E-53	1.35E-51
gene1726	5689.35	16170.80	2.84	MULTISPECIES: 3-isopropylmalate dehydratase large subunit	4.2E-120	1.5E-117
gene3469	4633.68	15383.76	3.32	MULTISPECIES: hypothetical protein	1.12E-12	9.42E-12
gene3850	5802.93	13933.83	2.40	dihydroxy-acid dehydratase	6.49E-67	5.61E-65
gene560	5445.92	13822.37	2.54	phosphate acetyltransferase	4.02E-47	1.87E-45
gene4130	5533.90	11799.86	2.13	iron-sulfur cluster repair di-iron protein	4.16E-24	7.26E-23
gene460	4524.79	11569.63	2.56	oxygen-independent coproporphyrinogen III oxidase	1.03E-63	8.28E-62
gene3665	3784.10	8944.60	2.36	TonB-dependent receptor	2.05E-40	7.48E-39
gene452	4120.60	8536.75	2.07	heavy metal translocating P-type ATPase metal-binding domain-containing protein	3.83E-57	2.46E-55

Statistical significance was determined using the DESeq2 package. *P* values were adjusted by Benjamini-Hochberg FDR correction ($n = 3$ biologically independent samples per group).

Table S12 Relative expression levels of significantly upregulated genes (top 5 genes with the greatest fold changes) in *F. anhuiense* K5 between the control and tocopherol acetate treatment groups. C and T represent the control and tocopherol acetate treatments, respectively.

Gene Id	Relative expression (FPKM)		Fold	Functinos	*P*	*P*_adj
	C	T				
gene1694	327.31	3449.62	10.54	Major facilitator superfamily	1.58E-96	3.04E-94
gene273	121.51	1162.69	9.57	MULTISPECIES: DUF3244 domain-containing protein	7.1E-173	3.8E-170
gene271	421.46	3497.97	8.30	MULTISPECIES: T9SS type A sorting domain-containing protein	1E-285	1.4E-282
gene272	3563.06	27451.68	7.70	secretion protein	0	0
gene2274	1.33	9.54	7.16	hypothetical protein	0.009751	0.022958
gene3636	30.14	179.65	5.96	MULTISPECIES: hypothetical protein	2.54E-13	2.25E-12
gene274	671.01	3820.22	5.69	MULTISPECIES: helix-turn-helix domain-containing protein	7.8E-189	4.7E-186

Statistical significance was determined using the DESeq2 package. *P* values were adjusted by Benjamini-Hochberg FDR correction ($n = 3$ biologically independent samples per group).

.

Table S13 Relative expression levels of significantly downregulated genes (top 15 genes with the greatest abundance) in *F. anhuiense* K5 between the control and tocopherol acetate treatments. C and T represent the control and tocopherol acetate treatments, respectively.

Gene Id	Relative expression (FPKM)		Fold	Functinos	*P*	*P*_adj
	C	T				
gene3323	148606.61	62423.21	0.42	SusC/RagA family TonB-linked outer membrane protein	3.31E-93	5.85E-91
gene614	44189.04	20570.36	0.47	gliding motility-associated C-terminal domain-containing protein	1.94E-71	1.95E-69
gene1833	32120.92	16043.64	0.50	MULTISPECIES: RagB/SusD family nutrient uptake outer membrane protein	1.42E-58	9.52E-57
gene3878	26927.43	12535.07	0.47	SusC/RagA family TonB-linked outer membrane protein	5.59E-70	5.26E-68
gene1978	25479.30	11312.35	0.44	MULTISPECIES: 50S ribosomal protein L21	1.71E-41	6.42E-40
gene279	18747.50	4497.69	0.24	MULTISPECIES: glutamine synthetase beta-grasp domain-containing protein	1.3E-215	1.1E-212
gene2489	15841.85	4767.09	0.30	MULTISPECIES: zinc metalloprotease	2.68E-84	3.55E-82
gene500	10760.36	3932.38	0.37	bifunctional aspartate kinase/homoserine dehydrogenase I	9.3E-108	2.5E-105
gene1835	11001.01	3516.60	0.32	T9SS type A sorting domain-containing protein	6.45E-52	3.6E-50
gene497	8834.01	3950.07	0.45	threonine synthase	3.27E-69	3.02E-67
gene616	7718.58	3104.58	0.40	OmpA family protein	5.73E-85	7.84E-83
gene4286	7733.69	1045.29	0.14	MULTISPECIES: alpha-amylase	0	0
gene4397	4796.52	2100.34	0.44	MULTISPECIES: MotA/TolQ/ExbB proton channel family protein	3.54E-63	2.68E-61
gene615	4849.44	1767.44	0.36	type IX secretion system membrane protein PorP/SprF	7.76E-41	2.89E-39
gene1844	4669.30	1911.33	0.41	peptidylprolyl isomerase	1.5E-46	6.7E-45

Statistical significance was determined using the DESeq2 package. *P* values were adjusted by Benjamini-Hochberg FDR correction ($n = 3$ biologically independent samples per group).

.

Table S14 Relative expression levels of significantly downregulated genes (top 5 genes with the greatest fold changes) in *F. anhuiense* K5 between the control and tocopherol acetate treatment groups. C and T represent the control and tocopherol acetate treatments, respectively.

Gene Id	Relative expression (FPKM)		Fold	Functinos	*P*	*P*_adj
	C	T				
gene4261	2630.52	261.50	0.10	SusC/RagA family TonB-linked outer membrane protein	1.2E-282	1.3E-279
gene4286	7733.69	1045.29	0.14	MULTISPECIES: alpha-amylase	0	0
gene4262	2009.70	276.77	0.14	SusD/RagB family nutrient-binding outer membrane lipoprotein	4.4E-202	3.1E-199
gene3024	571.01	90.16	0.16	protease complex subunit PrcB family protein	1.94E-78	2.29E-76
gene1852	90.55	15.98	0.18	MULTISPECIES: hypothetical protein	6.4E-12	5.07E-11
gene3025	159.93	28.61	0.18	hypothetical protein	4.22E-25	7.85E-24
gene694	957.64	178.51	0.19	glycoside hydrolase family 3 C-terminal domain-containing protein	4.27E-71	4.21E-69
gene1032	1638.74	323.64	0.20	class 1 fructose-bisphosphatase	9.5E-135	4E-132

Statistical significance was determined using the DESeq2 package. *P* values were adjusted by Benjamini-Hochberg FDR correction ($n = 3$ biologically independent samples per group).

.

Table S15 The control efficacy of treatments in planta assay

Treatment	Suppression efficacy (%)
T	51.68
K	52.92
TK	72.26

Table S16 Soil chemical properties

Total C (g kg^{-1})	Total N (g kg^{-1})	Available N (mg/kg)	Available P (mg/kg)	Available K (mg/kg)	pH
19.1±0.5	2.2±0.04	165.15±9.43	84.73±3.67	269.76±10.2	6.58±0.07

Table S17 Primers used in this study

Primer	Sequence (5'-3')	Reference
TBDT-F	TACTTCAGACGCTGGTAACG	This study
TBDT-R	AGAAGAAGCAAAATCGGGCA	
MFS-F	GCCGTAAGCTGGATGGTTA	This study
MFS-R	GCCAGAAACAGAGACGGAAT	
gyrA-F	GCAGTTGGTATGGCGACTAA	This study
gyrA-R	ACAATACGTCCTCTACCCGT	
Fusarium_tF	GGTTAGTCACTTTCCCTTCG	This study
Fusarium_tR	GACGCACTGATTGAGGTTGT	
Flavobacterium_seq1_F	AAATAATGAAGCAAGCCAAAGG	Qingping et al. (2021)
Flavobacterium_seq1_R	TGAATCCATAAAGCACGGAAA	

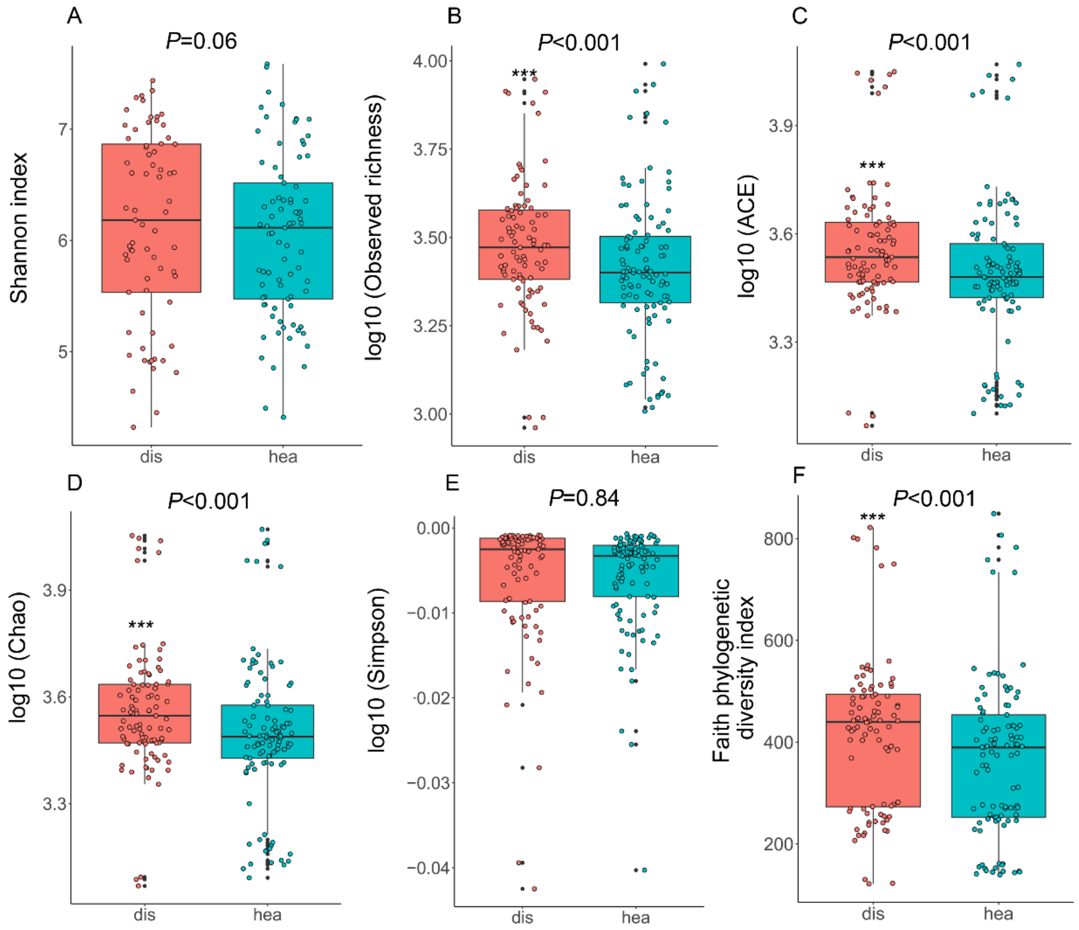

Fig. S1. Changes in alpha diversity caused by *Fusarium* infection in meta data

Statistical significance was determined based on a linear mixed effects model with the following formula: index ~ healthy or disease treatment + (1 | OAU). followed by ANOVA. Exact *P*-values: Shannon = 6.26×10^{-2} (A), Observed features= 9.88×10^{-4} (B), ACE = 7.84×10^{-4} (C), Chao = 8.37×10^{-4} (D), Faith's phylogenetic = 3.03×10^{-4} (F). Data are presented as box plots (center line, median; box limits, upper and lower quartiles (25th/75th percentiles); whiskers, min/max). All analyses were performed on n = 93 diseased and 105 healthy independent rhizosphere samples. ***$P < 0.001$, **$P < 0.01$, *$P < 0.05$.

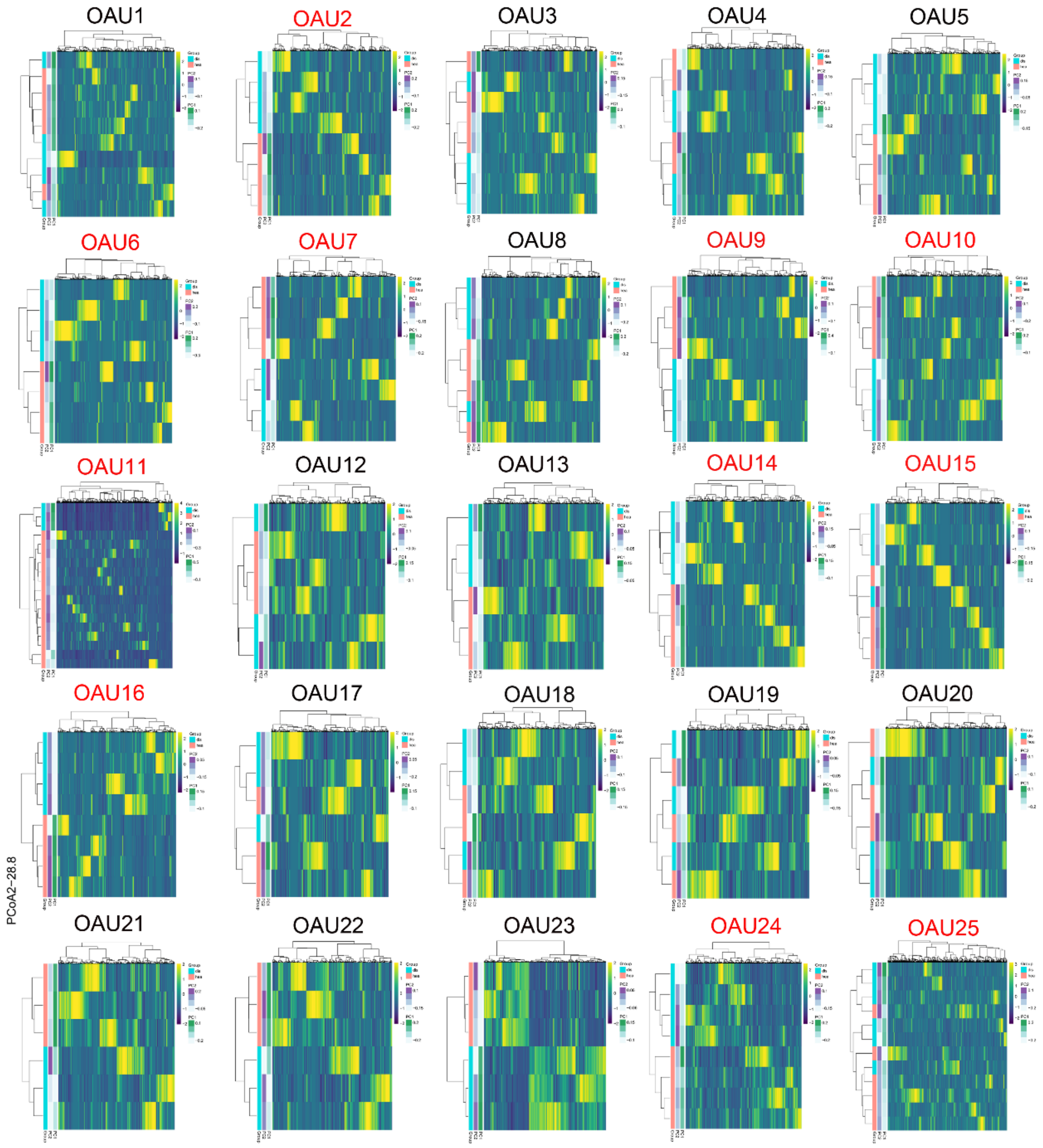

Fig. S2. Beta-diversity analysis of the rhizosphere bacterial community at ASV level in OAU (operational analysis units) based on weight UniFrac distance in OAU. The OAU represents the subgroups whose disease and healthy sample groups could be compared under the same conditions. The OAUs that contained significantly distinct clusters between diseased and healthy communities are indicated in red. Statistical significances between diseased and healthy communities in OAUs were determined based on the anosim function in the vegan package. All analyses were performed on n = 93 diseased and 105 healthy independent rhizosphere samples.

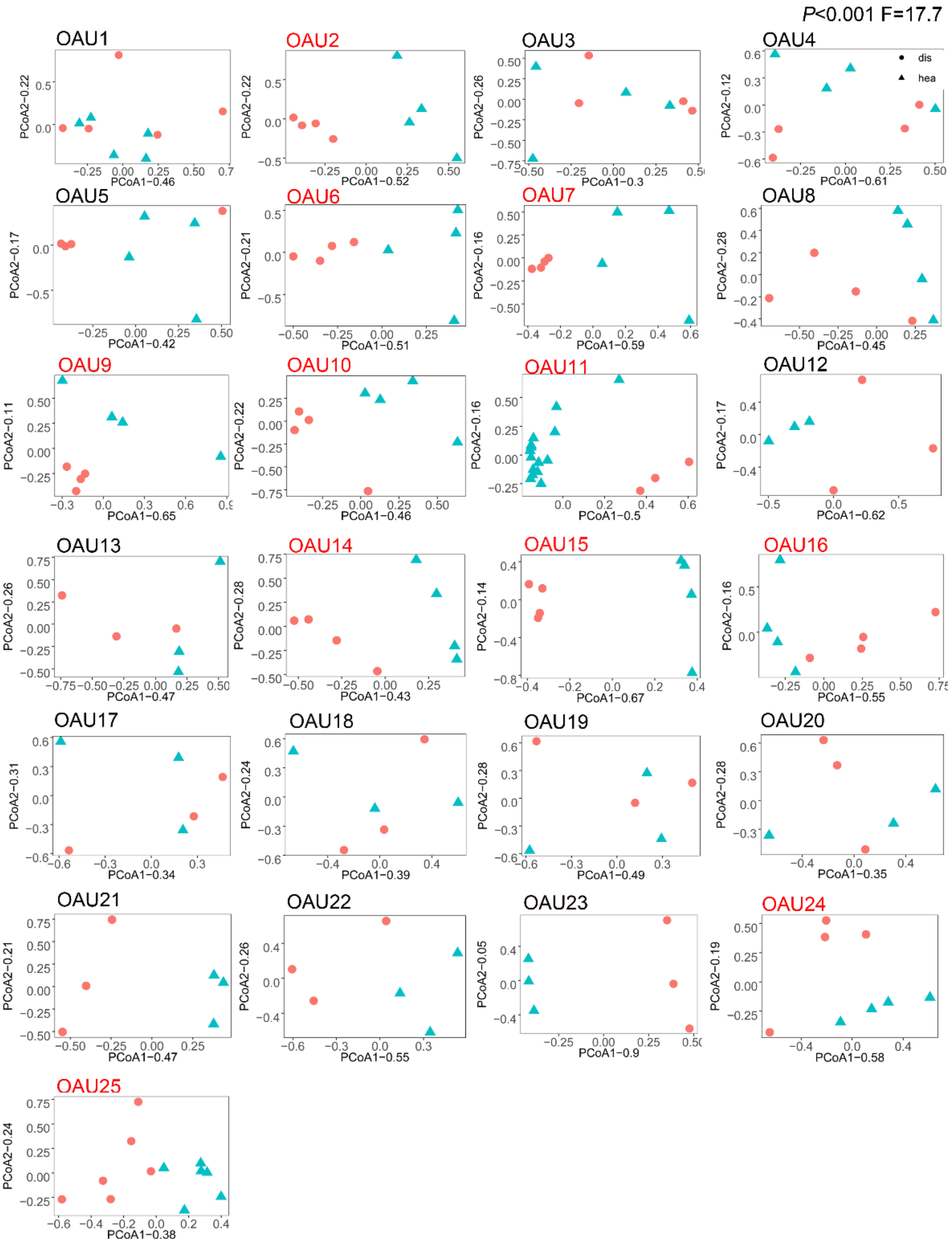

Fig. S3. Beta-diversity analysis of the rhizosphere bacterial community at genus level in OAU (operational analysis units) based on Bray-Curtis distance.

The OAU represents the subgroups whose disease and healthy sample groups could be compared under the same conditions. The OAUs that contained significantly distinct clusters between diseased and healthy communities are indicated in red. Statistical significances between diseased and healthy communities in OAUs were determined based on the anosim function in the vegan package. The combined analysis of community structure was based on a linear mixed effects model with the following formula: PcoA1 value ~ healthy or disease treatment + (1 | OAU), and the *P* value is located in the upper right corner of the figure. Exact *P*-value = 3.89×10^{-5}. All analyses were performed on $n = 93$ diseased and 105 healthy independent rhizosphere samples.

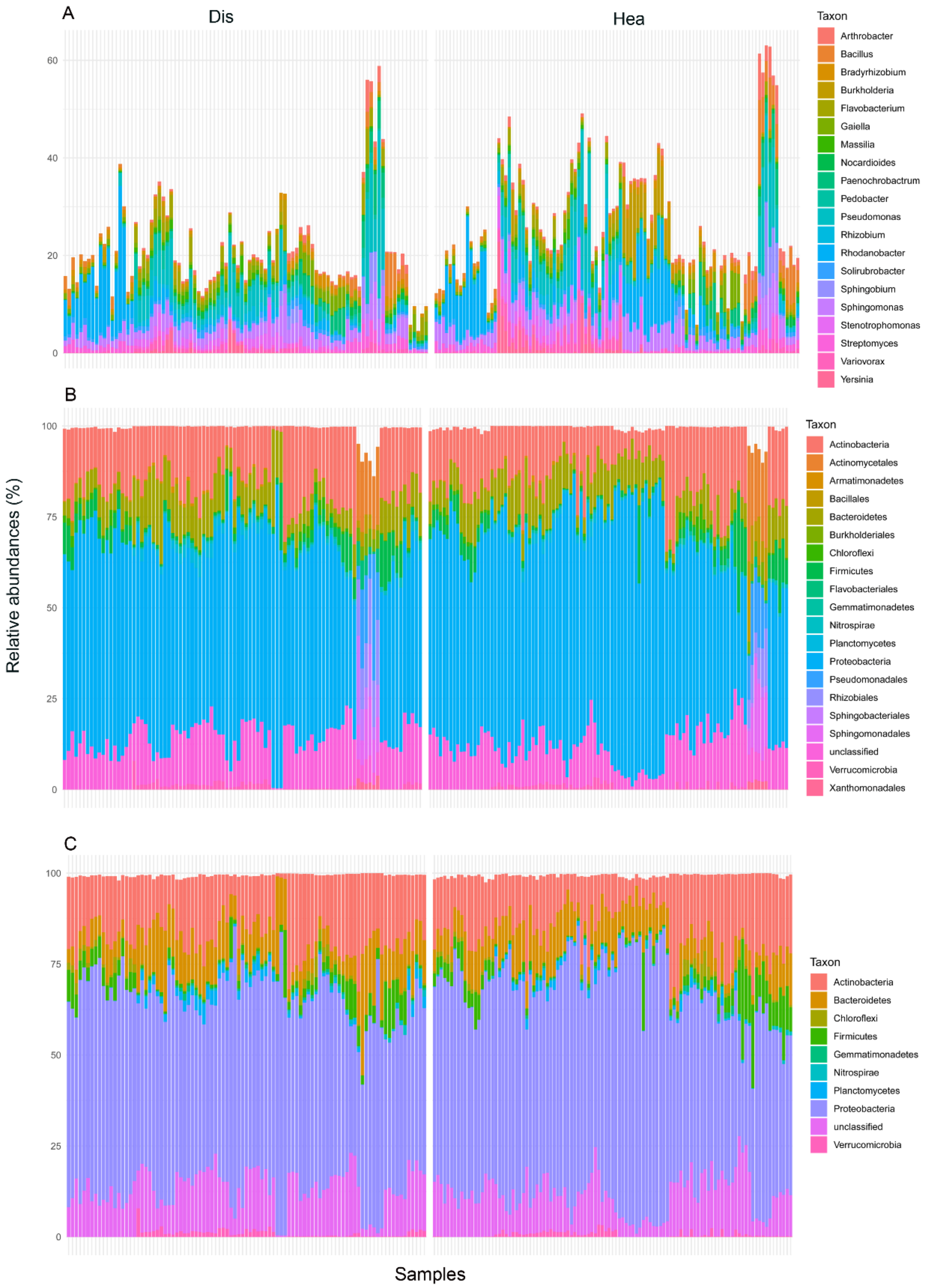

Fig. S4. Taxonomic composition comparison between diseased and healthy communities in rhizosphere at (A) genus (top 20), (B) order (top 20), and (C) phylum (top 10) levels. Relative abundances are shown as percentage of total sequences. All analyses were performed on n = 93 diseased and 105 healthy independent rhizosphere samples.

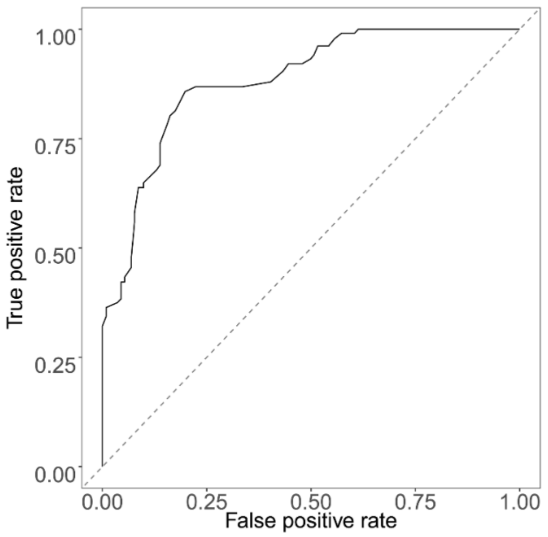

Fig. S5. Receiver operating characteristic (ROC) curve of the Random Forest classifier, showing the relationship between true positive rate (sensitivity) and false positive rate (1-specificity). The diagonal line represents random chance performance. All analyses were performed on n = 93 diseased and 105 healthy independent rhizosphere samples.

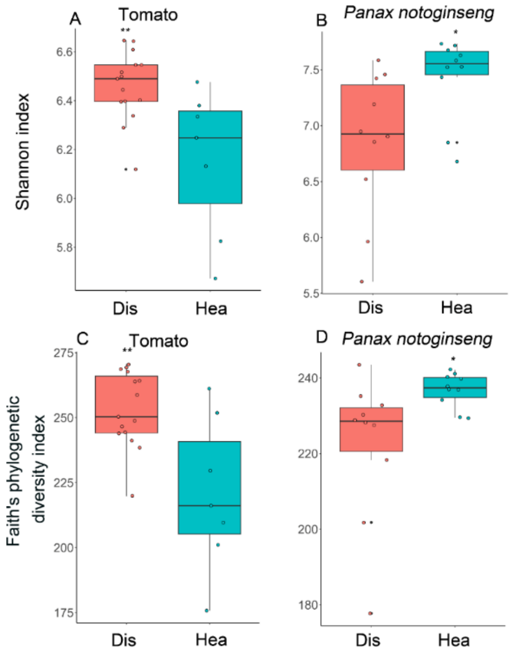

Fig. S6. Changes in alpha diversity caused by *Fusarium* infection in the rhizosphere bacterial community of local amplicon sequencing data. Statistical significance was determined based on two-sided unpaired t-test. *** $P < 0.001$, ** $P < 0.01$, * $P < 0.05$. Exact P-values: Shannon = 3.55×10^{-3} (A) and 2.23×10^{-2} (B), Faith's phylogenetic =2.32×10^{-3} (C) and 3.27×10^{-2} (D). Data are presented as box plots (center line, median; box limits, upper and lower quartiles (25th/75th percentiles); whiskers, min/max). Analysis in (A, C) was performed on n = 15 and 7 for diseased and healthy independent rhizosphere samples, and in (B, D) on n = 10 independent rhizosphere samples. ***$P < 0.001$, **$P < 0.01$, *$P < 0.05$.

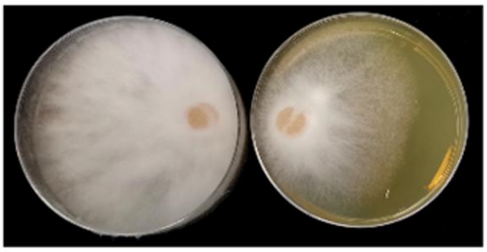

Fig. S7. Inhibition of the tomato pathogen *Fusarium oxysporum* by *Flavobacterium anhuiense* K5 whose 16S rRNA was mapped to ASV60 at the 97% similarity level.

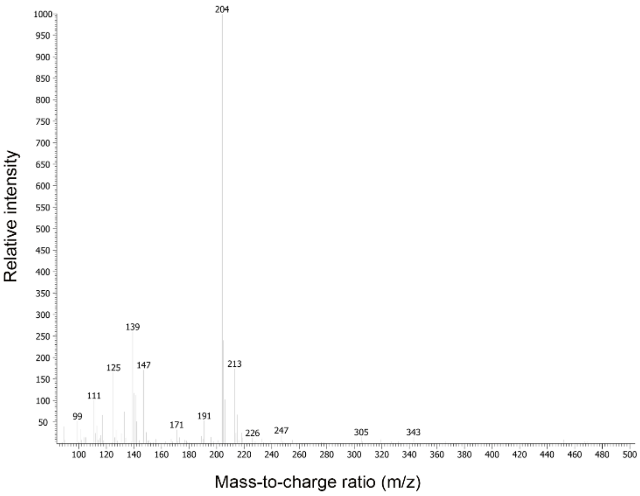

Fig. S8. MS/MS spectrum of tocopherol acetate

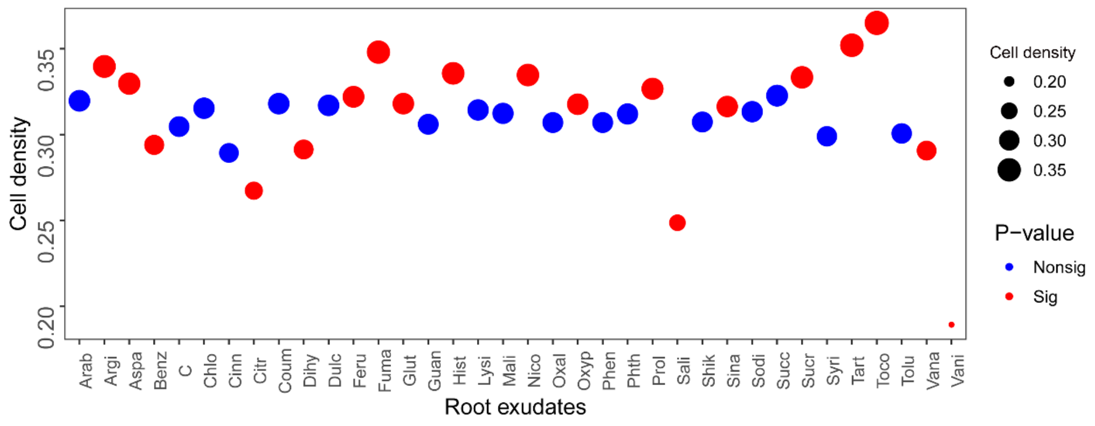

Fig. S9. The effects of 35 enriched root exudates on the growth of *F. anhuiense* K5 at 12 h (stationary phase). Circle size represents the cell density (OD600). Red and blue colors indicate components that significantly or non-significantly altered bacterial growth, respectively (Student's two-sided t-test, $n = 3$ biological replicates per condition). *** $P < 0.001$, ** $P < 0.01$, * $P < 0.05$.

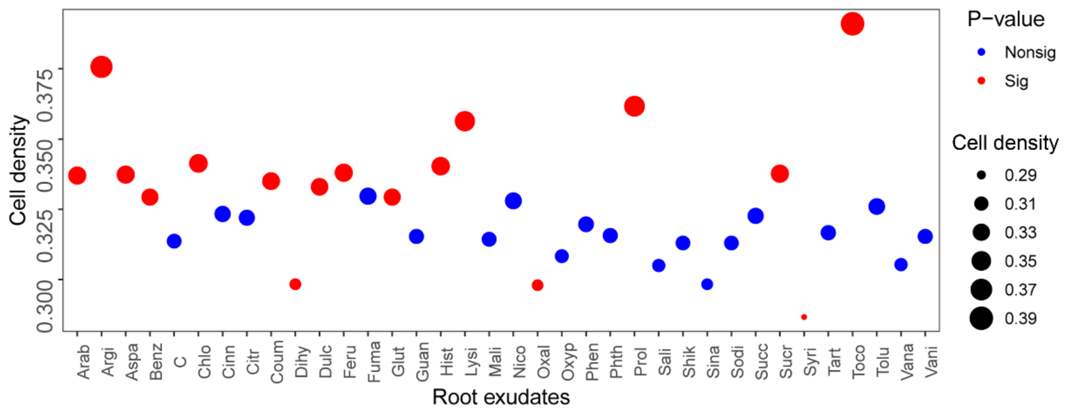

Fig. S10. The effects of 35 enriched root exudates on the growth of *F. anhuiense* K5 at 30h (stationary phase). Circle size represents the cell density (OD600). Red and blue colors indicate components that significantly or non-significantly altered bacterial growth, respectively (Student's two-sided t-test, $n = 3$ biological replicates per condition). *** $P < 0.001$, ** $P < 0.01$, * $P < 0.05$.

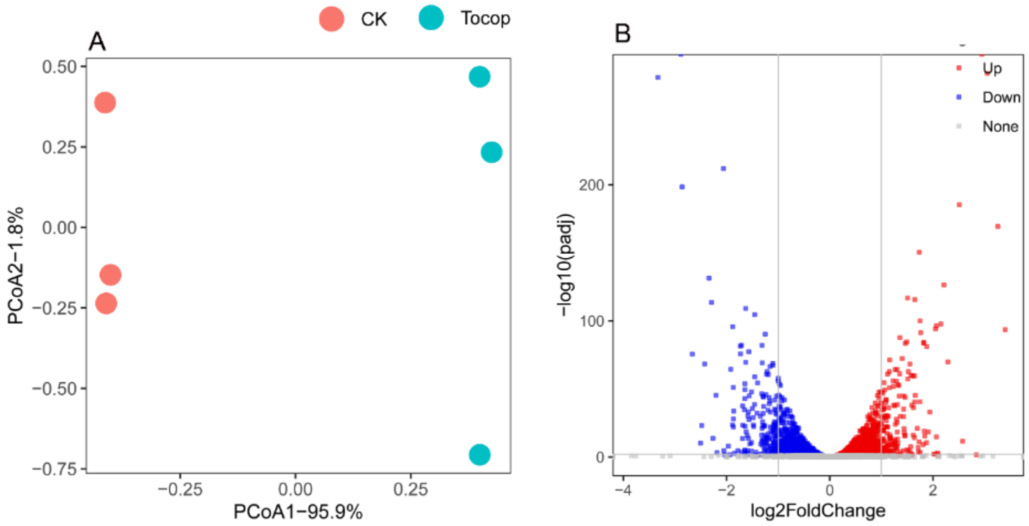

Fig. S11. Effects of tocopherol acetate on the transcriptome of *F. anhuiense* K5 ($n = 3$ biologically independent samples per group). Principal coordinate (A) and volcano plot (B) analyses of the transcriptome of *F. anhuiense* K. Statistical significance in (B) was determined based on a DESeq2 package (negative-binomial Wald test with Benjamini-Hochberg FDR correction).

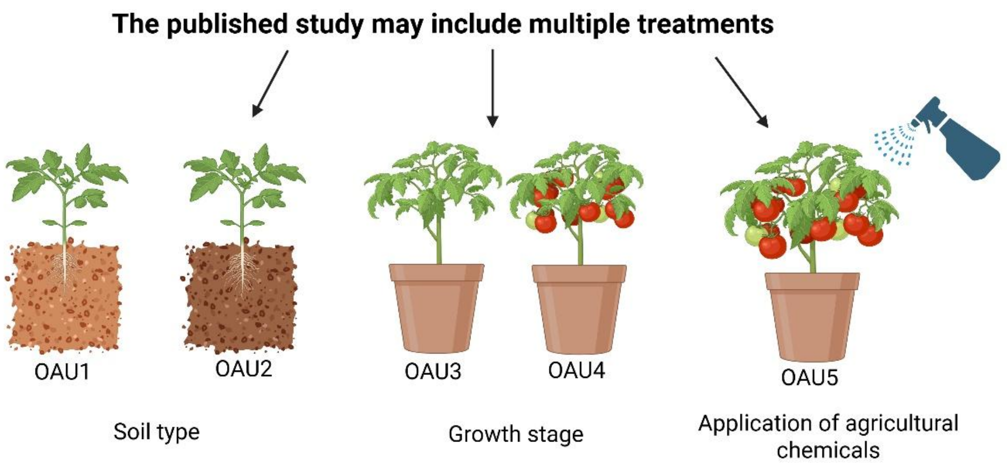

Fig. S12. The concept of OAU (operational analysis units).

Soil is an open environment where the microbiome is highly sensitive to external factors. Within a study, multiple treatments (e.g., different soil types or plant growth stages) may lead to significant variations in the rhizosphere microbiome, obscuring differences between healthy and diseased samples. To enable direct comparisons under consistent conditions (e.g., same soil and plant), we introduced Operational Analysis Units (OAUs). Samples from the same sampling location or time are grouped into one OAU, ensuring that healthy and diseased rhizosphere microbiomes are compared under identical environmental conditions. Created in BioRender. Lv, Su. (2025) https://www.biorender.com/l914k7v

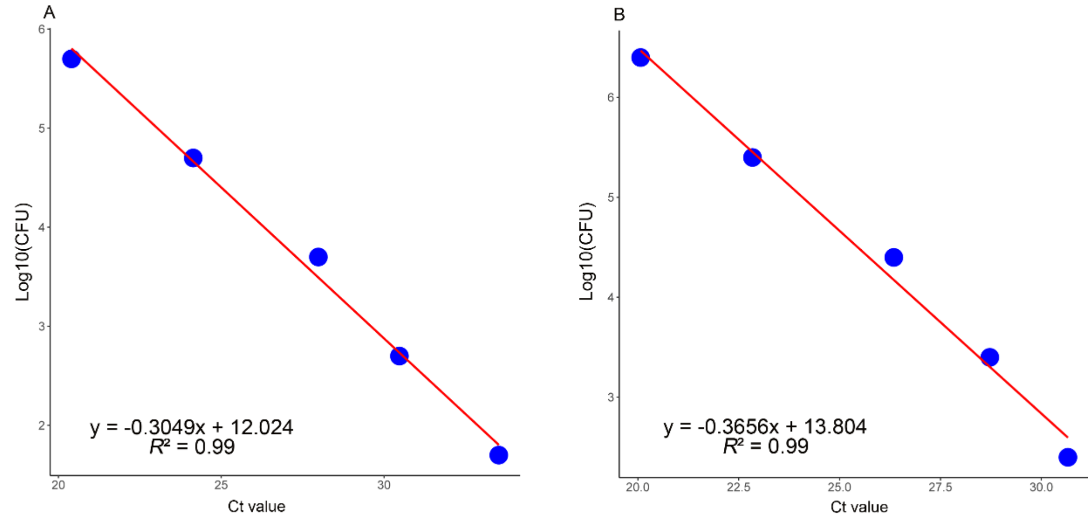

Fig. S13. The standard curves correlating Ct values with colony counts of *F. oxysporum* (A) and *F. anhuiense* (B). The regression lines were fitted using the lm function from the stats package in R. *P*-values for the regression models: A = 2.33×10^{-4}, B = 4.67×10^{-4}.

References

1. **Allemann, M.N., Kato, R., Carper, D.L., Hochanadel, L.H., Alexander, W.G., Giannone, R.J., Kamimura, N., Masai, E., and Michener, J.K.** (2025). Laboratory evolution in *Novosphingobium aromaticivorans* enables rapid catabolism of a model lignin-derived aromatic dimer. Appl Environ Microb **91,** e02081-02024.
2. **Ayala-Torres, A.M., Aranda-Ocampo, S., León-García de Alba, C.D., Nava-Díaz, C., and Sánchez-Pale, J.R.** (2023). Antagonistic bacteria against *Fusarium* spp. isolated from sclerotia of Claviceps gigantea in maize (Zea mays). Rev. mex. fitopatol **41,** 143-164.
3. **Fahde, S., Boughribil, S., Sijilmassi, B., and Amri, A.** (2023). Rhizobia: A promising source of plant growth-promoting molecules and their non-legume interactions: examining applications and mechanisms. Agriculture **13,** 1279.
4. **Finkel, O.M., Salas-González, I., Castrillo, G., Conway, J.M., Law, T.F., Teixeira, P.J.P.L., Wilson, E.D., Fitzpatrick, C.R., Jones, C.D., and Dangl, J.L.** (2020). A single bacterial genus maintains root growth in a complex microbiome. Nature **587,** 103-108.
5. **Fredendall, R.J., Stone, J.L., Pehl, M.J., and Orwin, P.M.** (2020). Transcriptome profiling of *Variovorax paradoxus* EPS under different growth conditions reveals regulatory and structural novelty in biofilm formation. Access Microbiol **2,** acmi000121.
6. **Hirsch, A.M., and Valdés, M.** (2010). Micromonospora: An important microbe for biomedicine and potentially for biocontrol and biofuels. Soil Boil Biochem **42,** 536-542.
7. **Kämpfer, P., Andersson, M.A., Rainey, F.A., Kroppenstedt, R.M., and Salkinoja-Salonen, M.** (1999). *Williamsia muralis gen.* nov., sp. nov., isolated from the indoor environment of a children's day care centre. Int J Syst Bacteriol **49,** 681-687.
8. **Kämpfer, P., Lipski, A., Lawrence, K.S., Olive, W.R., Newman, M.M., McInroy, J.A., and Viver, T.** (2025). *Flavobacterium plantiphilum* sp. nov., *Flavobacterium rhizophilum* sp. nov., *Flavobacterium rhizosphaerae* sp. nov., *Chryseobacterium terrae* sp. nov., and *Sphingomonas plantiphila* sp. nov. isolated from salty soil showing plant growth promoting potential. Syst Appl Microbiol **48,** 126588.
9. **Ling, L., Yue, R., Wang, Y., Feng, L., Yang, L., Li, Y., Mo, R., Zhang, W., Kong, F., Jiang, Y., and Zhou, Y.** (2024). Volatile organic compounds from *Stenotrophomonas geniculata* J-0 as potential biofumigants manage bulb rot caused by *Fusarium oxysporum* in postharvest Lanzhou lily. World J Microbiol Biotechnol **41,** 9.
10. **Liu, H., Li, J., Carvalhais, L.C., Percy, C.D., Prakash Verma, J., Schenk, P.M., and Singh, B.K.** (2021). Evidence for the plant recruitment of beneficial microbes to suppress soil-borne pathogens. New Phytol **229,** 2873-2885.
11. **Liu, Y., Wang, H., Qian, X., Gu, J., Chen, W., Shen, X., Tao, S., Jiao, S., and Wei, G.** (2023). Metagenomics insights into responses of rhizobacteria and their alleviation role in licorice allelopathy. Microbiome **11,** 109.
12. **Qingping, W., Baoqing, Z., Qinghua, Y., Zhenjie, L., Fan, L., Yuting, S., Xinran, X., Chufang, W., Jumei, Z., and Yu, D.** (2021). Molecular targets and quantitative detection methods for screening *Flavobacterium*. CN Patent CN113373249B, 2022.
13. **Wang, Y., Xie, T., Ma, C., Zhao, Y., Li, J., Li, Z., and Ye, X.** (2024). Biochemical characterization and antifungal activity of a recombinant β-1,3-glucanase FlGluA from *Flavobacterium* sp. NAU1659. Protein Expr Purif **224**, 106563.
