# Supplementary material for: General variation in the Fusarium wilt rhizosphere microbiome
Source: Nat Commun. 2025 Dec 27;17:1017. doi: 10.1038/s41467-025-67760-y (PMC12847888; doi:10.1038/s41467-025-67760-y)
Supplement: Supplementary file 2 — Description of Additional Supplementary Files [file 41467_2025_67760_MOESM2_ESM.pdf]

Description of Additional Supplementary Files

File Name: Supplementary Data 1

Description: The projects retrieved in this study.

File Name: Supplementary Data 2

Description: Significance test of principal coordinate analysis based on weight UniFrac distance at AVS level in each OAU.

File Name: Supplementary Data 3

Description: Significance test of principal coordinate analysis based on Bray-Curtis distance at genus level in each OAU.

File Name: Supplementary Data 4

Description: Published studies related to root exudates of Fusarium-infected plant.

File Name: Supplementary Data 5

Description: The enriched root exudates from Fusarium-infected plants.
