# Supplementary material for: General variation in the Fusarium wilt rhizosphere microbiome
Source: Nat Commun. 2025 Dec 27;17:1017. doi: 10.1038/s41467-025-67760-y (PMC12847888; doi:10.1038/s41467-025-67760-y)
Supplement: Supplementary file 8 — Reporting Summary [file 41467_2025_67760_MOESM8_ESM.pdf]

nature portfolio

Corresponding author(s): Ruifu Zhang; Yongliang Yan

Last updated by author(s): Dec 5, 2025

Reporting Summary

Nature Portfolio wishes to improve the reproducibility of the work that we publish. This form provides structure for consistency and transparency in reporting. For further information on Nature Portfolio policies, see our Editorial Policies and the Editorial Policy Checklist.

Statistics

For all statistical analyses, confirm that the following items are present in the figure legend, table legend, main text, or Methods section.

n/a	Confirmed	
☐	☒	The exact sample size (*n*) for each experimental group/condition, given as a discrete number and unit of measurement
☐	☒	A statement on whether measurements were taken from distinct samples or whether the same sample was measured repeatedly
☐	☒	The statistical test(s) used AND whether they are one- or two-sided<br>*Only common tests should be described solely by name; describe more complex techniques in the Methods section.*
☐	☒	A description of all covariates tested
☐	☒	A description of any assumptions or corrections, such as tests of normality and adjustment for multiple comparisons
☐	☒	A full description of the statistical parameters including central tendency (e.g. means) or other basic estimates (e.g. regression coefficient) AND variation (e.g. standard deviation) or associated estimates of uncertainty (e.g. confidence intervals)
☐	☒	For null hypothesis testing, the test statistic (e.g. *F*, *t*, *r*) with confidence intervals, effect sizes, degrees of freedom and *P* value noted<br>*Give P values as exact values whenever suitable.*
☒	☐	For Bayesian analysis, information on the choice of priors and Markov chain Monte Carlo settings
☐	☒	For hierarchical and complex designs, identification of the appropriate level for tests and full reporting of outcomes
☐	☒	Estimates of effect sizes (e.g. Cohen's *d*, Pearson's *r*), indicating how they were calculated

Our web collection on statistics for biologists contains articles on many of the points above.

Software and code

Policy information about availability of computer code

Data collection	PubMed, Google Scholar, and the NCBI Sequence Read Archive (SRA) were used for data collection
Data analysis	Raw sequences were analyzed in vsearch, MEGAHIT, and R software.

For manuscripts utilizing custom algorithms or software that are central to the research but not yet described in published literature, software must be made available to editors and reviewers. We strongly encourage code deposition in a community repository (e.g. GitHub). See the Nature Portfolio guidelines for submitting code & software for further information.

Data

Policy information about availability of data

All manuscripts must include a data availability statement. This statement should provide the following information, where applicable:

- Accession codes, unique identifiers, or web links for publicly available datasets
- A description of any restrictions on data availability
- For clinical datasets or third party data, please ensure that the statement adheres to our policy

The raw sequence data were deposited in the SRA under accession number PRJNA1138403, PRJNA1240447 and PRJNA1240449.

Research involving human participants, their data, or biological material

Policy information about studies with human participants or human data. See also policy information about sex, gender (identity/presentation), and sexual orientation and race, ethnicity and racism.

Reporting on sex and gender	n/a
Reporting on race, ethnicity, or other socially relevant groupings	n/a
Population characteristics	n/a
Recruitment	n/a
Ethics oversight	n/a

Note that full information on the approval of the study protocol must also be provided in the manuscript.

Field-specific reporting

Please select the one below that is the best fit for your research. If you are not sure, read the appropriate sections before making your selection.

☐ Life sciences ☐ Behavioural & social sciences ☒ Ecological, evolutionary & environmental sciences

For a reference copy of the document with all sections, see nature.com/documents/nr-reporting-summary-flat.pdf

Ecological, evolutionary & environmental sciences study design

All studies must disclose on these points even when the disclosure is negative.

Study description	To evaluate Fusarium wilt effects on rhizosphere microbiota, we analyzed amplicon data from published studies and conducted validation experiments with tomato/Panax notoginseng (amplicon sequencing + root exudate metabolomics).
Research sample	Our integrated analysis comprised: (1) amplicon sequencing data from 198 rhizosphere samples (93 diseased, 105 healthy) across 18 locations and seven host species (pea, Panax, pepper, tomato, avocado, watermelon, cucumber); and (2) metagenomic data from 22 samples (11 diseased/healthy) spanning three locations (Panax and Zanthoxylum hosts). For experimental validation, we collected rhizosphere soils from randomly selected plants (tomato: 15 treated + 7 controls; P. notoginseng: 10 diseased + 10 healthy individuals).
Sampling strategy	We systematically analyzed Fusarium-induced changes in rhizosphere bacterial communities through: 1) a comprehensive literature review of PubMed, Google Scholar, and NCBI SRA databases using strict inclusion/exclusion criteria (Methods), and 2) validation experiments with tomato/Panax notoginseng plant samples. This dual approach ensured both broad data coverage and methodological rigor.
Data collection	Our meta-analysis systematically identified studies through PubMed, Google Scholar, and NCBI SRA using Fusarium-rhizosphere search terms. Inclusion required: (1) amplicon sequencing data of rhizosphere microbiomes under Fusarium infection, (2) detailed methodological documentation, and (3) ≥6 samples with complete metadata. Disease status was defined by original studies' treatment classifications. For experimental validation, we collected both pathogen-inoculated (diseased) and untreated (healthy) plant samples
Timing and spatial scale	The data was collected between 2021.6 and 2021.12, spanning a total of 28 regions. These samples were obtained from 7 plants (pea, panax, pepper, tomato, avocado, watermelon and cucumber). For experimental validation, additional tomato and P. notoginseng samples were collected in December 2024.
Data exclusions	Our systematic search identified 37 amplicon and 4 metagenomic studies, from which we excluded 7 fungal-focused amplicon studies due to insufficient metadata. After further quality filtering, 15 amplicon studies were retained (6 excluded for sequence QC failures, 9 for incomplete metadata, and 6 for non-Fusarium pathogens), while all 4 metagenomic studies met inclusion criteria. For experimental validation, complete datasets were analyzed without exclusions.
Reproducibility	Regarding the verification of the enrichment of Flavobacterium in the diseased rhizosphere, we conducted an analysis of previously reported literature. Additionally, we performed local, limited-scale experiments to validate this finding in both ginseng and tomato plants.
Randomization	Disease status classification followed the original studies' criteria for meta-analysis data. For validation experiments, plants inoculated with Fusarium pathogens were designated as diseased treatment, while untreated plants served as healthy controls
Blinding	Blinding was not applied because: (1) microbial community data were generated through high-throughput sequencing (objective measurement); (2) alpha/beta diversity analyses were based on standardized bioinformatics pipelines; and (3) blinding is uncommon in microbiome studies where outcomes are instrument-derived

Did the study involve field work? ☒ Yes ☐ No

Field work, collection and transport

Field conditions	The study site is situated at Miaoxiang Sanqi Technology Co., Ltd., in the Wenshan County of Yunnan Province, the geo-authentic region for the production of Sanqi ginseng in China. This site has a yellow-brown soil derived from pelite and/or carbonolite and is classified as Alfisols based on the Chinese soil taxonomy. The average annual radiation hours are 1500–2000 h and the average relative humidity is 75–85%. This region has a subtropical climate with an average annual temperature of 15–17 °C and an annual frost-free period of 300 days. The average annual precipitation is 1100–1319 mm, of which nearly 60% falls in the humid season (June to August), and 40% in the dry season (September to May).
Location	Wenshan County of Yunnan Province (23°34′N, 104°19′E, 1500 m alt.)
Access & import/export	All samples were collected from cultivated fields in Wenshan County, Yunnan Province, China—a geo-authentic production area for Panax notoginseng. The collection was conducted with explicit permission from the landowner(s) and did not involve protected areas, endangered species, or cross-border transport. As this study exclusively utilized cultivated plants from private agricultural land, no additional permits from local, national, or international authorities were required
Disturbance	This study involved the collection of a small number of samples exclusively from cultivated Panax notoginseng plantations in Wenshan County. Due to the controlled agricultural setting and minimal sampling scale, no significant ecological disturbance was caused.

Reporting for specific materials, systems and methods

We require information from authors about some types of materials, experimental systems and methods used in many studies. Here, indicate whether each material, system or method listed is relevant to your study. If you are not sure if a list item applies to your research, read the appropriate section before selecting a response.

Materials & experimental systems

n/a	Involved in the study	
☒	☐	Antibodies
☒	☐	Eukaryotic cell lines
☒	☐	Palaeontology and archaeology
☒	☐	Animals and other organisms
☒	☐	Clinical data
☒	☐	Dual use research of concern
☐	☒	Plants

Methods

n/a	Involved in the study	
☒	☐	ChIP-seq
☒	☐	Flow cytometry
☒	☐	MRI-based neuroimaging

Dual use research of concern

Policy information about dual use research of concern

Hazards

Could the accidental, deliberate or reckless misuse of agents or technologies generated in the work, or the application of information presented in the manuscript, pose a threat to:

No	Yes	
☒	☐	Public health
☒	☐	National security
☒	☐	Crops and/or livestock
☒	☐	Ecosystems
☒	☐	Any other significant area

Experiments of concern

Does the work involve any of these experiments of concern:

No	Yes	
☒	☐	Demonstrate how to render a vaccine ineffective
☒	☐	Confer resistance to therapeutically useful antibiotics or antiviral agents
☒	☐	Enhance the virulence of a pathogen or render a nonpathogen virulent
☒	☐	Increase transmissibility of a pathogen
☒	☐	Alter the host range of a pathogen
☒	☐	Enable evasion of diagnostic/detection modalities
☒	☐	Enable the weaponization of a biological agent or toxin
☒	☐	Any other potentially harmful combination of experiments and agents

Plants

Seed stocks	Tomato seeds ('hezuo 903') were obtained from Jiangsu Academy of Agricultural Sciences. For Panax notoginseng, rhizosphere soil samples were collected from a geo-authentic plantation in Wenshan County, Yunnan (23°34′N, 104°19′E; 1500 m altitude).
Novel plant genotypes	n/a
Authentication	Seed authenticity was verified through quality control procedures conducted by the original manufacturer/supplier
